# Purifying Selection in Deeply Conserved Human Enhancers Is More Consistent than in Coding Sequences

**DOI:** 10.1371/journal.pone.0103357

**Published:** 2014-07-25

**Authors:** Dilrini R. De Silva, Richard Nichols, Greg Elgar

**Affiliations:** 1 Systems Biology, MRC National Institute for Medical Research, Mill Hill, London, United Kingdom; 2 School of Biological and Chemical Sciences, Queen Mary University of London, London, United Kingdom; University of Iceland, Iceland

## Abstract

Comparison of polymorphism at synonymous and non-synonymous sites in protein-coding DNA can provide evidence for selective constraint. Non-coding DNA that forms part of the regulatory landscape presents more of a challenge since there is not such a clear-cut distinction between sites under stronger and weaker selective constraint. Here, we consider putative regulatory elements termed Conserved Non-coding Elements (CNEs) defined by their high level of sequence identity across all vertebrates. Some mutations in these regions have been implicated in developmental disorders; we analyse CNE polymorphism data to investigate whether such deleterious effects are widespread in humans. Single nucleotide variants from the HapMap and 1000 Genomes Projects were mapped across nearly 2000 CNEs. In the 1000 Genomes data we find a significant excess of rare derived alleles in CNEs relative to coding sequences; this pattern is absent in HapMap data, apparently obscured by ascertainment bias. The distribution of polymorphism within CNEs is not uniform; we could identify two categories of sites by exploiting deep vertebrate alignments: stretches that are non-variant, and those that have at least one substitution. The conserved category has fewer polymorphic sites and a greater excess of rare derived alleles, which can be explained by a large proportion of sites under strong purifying selection within humans – higher than that for non-synonymous sites in most protein coding regions, and comparable to that at the strongly conserved trans-dev genes. Conversely, the more evolutionarily labile CNE sites have an allele frequency distribution not significantly different from non-synonymous sites. Future studies should exploit genome-wide re-sequencing to obtain better coverage in selected non-coding regions, given the likelihood that mutations in evolutionarily conserved enhancer sequences are deleterious. Discovery pipelines should validate non-coding variants to aid in identifying causal and risk-enhancing variants in complex disorders, in contrast to the current focus on exome sequencing.

## Introduction

The effect of mutations in coding sequence is a well-characterised phenomenon whereby non-synonymous changes alter the resulting protein product. Therefore, these non-synonymous changes tend to be under stronger purifying selection than synonymous mutations. There are only rare instances where synonymous changes have been demonstrated to have phenotypic effects [1]. This difference can be exploited in the analysis of genomes; for example, since synonymous changes are assumed to be largely neutral in effect, their genetic diversity can be used as a null distribution against which to test for evidence to identify loci at which non-synonymous changes have been established by positive selection (e.g. Macdonald-Kreitman test, [2]). Conversely, non-synonymous changes are treated as being more likely to be the cause of an altered phenotype. Indeed, genome-wide scans for causative mutations often concentrate on exons (the ‘exome’) and have been successful in detecting non-synonymous changes responsible for numerous genetic diseases, [3]. By contrast, there is scant data on the effect of mutations in regulatory sequences; in part because regulatory regions can be difficult to identify. Nevertheless, a few studies have been able to identify *cis*-regulatory mutations implicated in human diseases. For example, beta thalassemia and haemophilia B are both instances of human diseases caused by mutations affecting transcription-factor binding sites in regulatory sequences (reviewed in [4]). However, not all transcription-factor binding motifs are well characterised, and other types of sequence may also have regulatory activity. In these cases evolutionary conservation provides a means to identify sequences of functional importance to the organism. If a non-coding sequence has been conserved across large evolutionary periods so that it is found in a number of diverse species, then this is most likely to be a consequence of selection against mutations that would modify it. Occasionally such mutations have been identified within a species and are indeed found to be deleterious. For example Lettice et al. found several heterozygous dominant point mutations in a highly conserved enhancer 1 Mb away from the Shh locus in humans that segregate with pre-axial polydactyly [5]. Similarly, Benko et al, found a heterozygous point mutation in a highly conserved non-coding element flanking the SOX9 locus that alters binding of the transcription factor MSX1 associated with the Pierre Robin syndrome [6]. More recently, two rare variants in the 5′ UTR and an intron of the *RBM8A* gene were found to segregate in individuals with Thrombocytopenia (reduced platelet count) with Absent Radii (TAR) syndrome when no exonic mutations were found in affected individuals at that locus [7].

Generally it has only been when exome sequencing fails to identify any causative mutations that non-coding DNA is surveyed. Now that whole genomes are being sequenced in greater numbers, such as in the 1000 Genomes Project [8], there is the opportunity to identify more non-coding variants. However, there are still hurdles to assessing their importance. Firstly large re-sequencing projects have tended to neglect non-exonic regions resulting in much lower coverage. Consequently, the stringent quality control measures used in SNP calling from next-generation sequencing data mean that rare variants in non-coding DNA may be filtered out as putative sequencing errors. Secondly, it is much harder to predict the consequence of mutation/variation in regulatory sequences because their grammar is poorly understood. An effective way of identifying putative regulatory sequences, in particular those that are under strong selection, has been to use cross species comparisons, often across large evolutionary distances - a method referred to as phylogenetic footprinting [9]. The various methods used to predict *cis*-regulatory modules often depend on a signal of evolutionary conservation [10], [11], [12]. In contrast, phylogenetic shadowing [13] using species that are more closely related helps identify regions that have diverged recently, and may have acquired a lineage-specific role.

Here, we focus our efforts on sequences that are conserved across all jawed vertebrates and that likely define a set of developmental regulatory elements (57 of the 1809 CNEs in this dataset have been tested functionally for enhancer activity in zebrafish and the data is available on the CONDOR website [14]). This core set of conserved non-coding elements (CNEs) was identified through multiple alignments of mammalian and Fugu genomes [14]. CNEs differ from other sets of conserved non-coding sequence that have been identified by comparative analyses in not overlapping known exons (e.g. Ultra-Conserved Elements identified by human-mouse-rat genomic comparisons [15] and Highly Conserved Elements [16]). About 80% of CNEs tested experimentally in zebrafish embryos show tissue-specific enhancer activity at various stages of embryonic development. 23 out of 25 CNEs tested from four different loci drive expression in zebrafish embryos [17] including SOX21 and PAX6-associated CNEs directing GFP-expression in the developing eye. A second study found 8 out of 10 elements drive reproducible reporter expression in the developing embryo [18]. The enrichment of vertebrate CNEs for conserved binding site motifs such as the Pbx-Hox heterodimer [19] and the over-representation of several transcription factor position weight matrices in mammalian conserved non-coding sequences [20] suggest that conservation of non-coding sequences is likely to be due, at least in part, to the presence of common transcription factor binding sites. Some transcription factors are highly sequence-specific and only bind to genomic regions with the exact transcription factor binding sequence [21]. In such highly specific interactions, any variation in the transcription factor binding sequence will have an effect on the transcription factor binding and subsequent gene expression, as seen with allele-specific binding of CTCF [22]. Conversely, those positions that are less strongly conserved may be less important for the functioning of the sequences. This logic is used in the construction of the Position Weight Matrix (PWM), which reflects the affinity of transcription factors to their preferred binding sites [23].

On average CNEs are about 200 bp in length (the maximum being *c.* 800 bp), yet their conservation cannot be explained by our current knowledge of transcription-factor binding sites, since most known binding targets are only 4-10 bp long. In fact the rate of evolution of known binding sites is faster than that of CNEs. One possibility would be if the binding sites overlap each other and the order of overlap is necessary to retain the proper function of the *cis*-regulatory module (as discussed in [24]). Another hypothesis is that conserved non-coding sequences (CNSs) represent mutational ‘coldspots’, however this explanation has been rejected [25] because of the excess of rare derived alleles observed within CNSs relative to polymorphisms outside CNSs that cannot be explained by population bottleneck effects or background selection. CNEs can be defined by a large number of completely (evolutionarily) conserved sites (Non Variable Regions), as well as a number of more variable sites (Restricted Variable Regions) based on their conservation across seven divergent vertebrate species. We evaluate the hypothesis that restricted variable regions in CNEs have been accumulating substitutions in the human lineage due to relaxed evolutionary constraint, resulting in more within-species polymorphism than non-variable regions. Using the occurrence and allele frequencies of SNPs from both the HapMap and 1000 Genomes Projects in CNEs we show evidence that a) non-variable regions within CNEs are under stronger selective constraint than restricted variable regions, b) the distribution of selective effects in CNEs is comparable to that in non-synonymous sites and c) there are discrepancies between the results obtained from HapMap and 1000 Genomes Project datasets.

## Results and Discussion

We compared the proportion of polymorphic sites and the derived allele-frequency spectra of single nucleotide polymorphisms (SNPs) in CNEs with other regions of the human genome, for the purpose of exploring selective constraint in CNEs, and in a wider context understanding the evolutionary forces that define large *cis*-regulatory modules in vertebrate genomes. Comparisons were made with three categories of region; non-synonymous sites (those resulting in amino acid changes), synonymous sites from coding regions, and non-exonic sequences that do not overlap any CNEs or other annotated regulatory features. The non-synonymous sites act as a positive control, since it is known that a subset of non-synonymous changes is counteracted by relatively strong purifying selection [26]. Conversely, on the assumption that they are under negligible selection, we have used synonymous and non-coding sites as our negative controls. We obtained SNPs that map to CNEs, coding and non-coding regions from the public databases of both the HapMap Project and the 1000 Genomes Project and used the derived allele frequencies of SNPs to compare selective constraint in these sequences. We built alignments of CNEs from the human, macaque, mouse, chicken, frog, zebrafish and fugu genomes and defined two categories of sites in CNEs after masking the human sequence from the alignment. The human sequences were masked to avoid an ascertainment bias, since the second step of the analysis uses human data to identify polymorphic sites. These species last shared a common ancestor over 450 million years ago and in total, the divergence between these species represents approximately 1.9 billion years of evolution during which CNEs have retained a high degree of sequence similarity (divergence times to common ancestor [27] are given in Table 1). CNE sites that are invariant across all six species (i.e. excluding human) in our alignments are defined as Non-Variable Regions (hereafter referred to as NVRs) and sites where at least one substitution is present in any of the species are called Restricted Variable Regions (hereafter referred to as RVRs). By excluding the human reference sequence in our definition of NVRs and RVRs, we avoided mis-classifying rapidly evolving human sites that are represented by the derived allele in the human reference genome. In reality, given that the human reference genome is generated from a consensus of a number of individuals, that we have also included another primate (macaque) in the alignments and that CNEs in general appear to be under strong and deep evolutionary constraint, the number of instances where a human site alone would change the definition from NVR to RVR is very small (see Methods). Conservation of sequences across such large divergence times indicates strong historical evolutionary constraint, most likely reflecting the importance of such sequences as functional elements. The proportion of polymorphic sites found in 1,809 CNEs spanning a length of 318,286 bp together with coding and non-coding regions are given in Table 2.

**Table 1:** Divergence time since last common ancestor.

Organism	Divergence time since last common ancestor (Mya)
Macaque	29.2
Mouse	92.4
Chicken	301.7
Frog	371.2
Zebrafish	400.1
Fugu	400.1

The divergence time since last common ancestor with human obtained from Time Tree (Hedges et al., 2006).

**Table 2:** Variants from 1000 Genomes Low Coverage Data across the different categories.

		DAC > = 1		DAC > = 6	
Type of site	No. of sites surveyed	No. of SNPs	% sites with SNPs	No. of SNPs	% sites with SNPs
**CNEs (Total)**	318286	3182	1.00	1119	0.35
a) NVR	161361	1259	0.78	400	0.25
b) RVR	156925	1923	1.22	719	0.46
**Coding sequences (Total)**	680811	6118	0.9	2340	0.34
c) Non-synonymous (64%)	437283	3492	0.79	1161	0.26
d) Synonymous (36%)	243528	2626	1.07	1179	0.48
**Non-coding**	398240	5259	1.32	2429	0.6

The proportion of sites that is polymorphic in each category using SNPs where at least one derived allele is reported (Derived Allele Count (DAC) > = 1) and those SNPs where at least six derived alleles are reported (DAC > = 6).

### Reduced diversity in CNEs is not due to a bias in variant calling

From Table 2 we observe a reduced frequency of variants in CNEs relative to synonymous sites suggesting that mutations in CNEs are subject to continuing purifying selection in the human genome. Although the 1000 Genomes Project uses a whole genome approach, the Variant Quality Score Recalibrator algorithm used by GATK to call SNPs relies on HapMap 3 sites to build the Gaussian Mixture model used to classify a variant as a “true site” [28]. This could mean that fewer SNPs in non-coding regions are being reported, resulting in the low levels of variation we observe in CNEs. If this was indeed the case, then the same bias should extend to non-conserved non-coding sites. However, the proportion of CNE sites that are polymorphic is significantly lower than that at non-conserved non-coding sites demonstrating that the observed low levels of variation at CNE sites is not an artefact of the variant calling procedure. Polymorphism is also significantly lower at synonymous sites compared with non-conserved non-coding sites. This trend is expected under a model of neutral evolution, since the synonymous sites are closely linked to non-synonymous sites that can experience either positive or purifying selection. The consequent effects of hitchhiking and background selection depress effective population size and hence polymorphism in coding regions [29], along with other influences such as the effects of epigenetic modification [30].

### Sites within CNEs are subject to different levels of constraint

**Figure 1:**
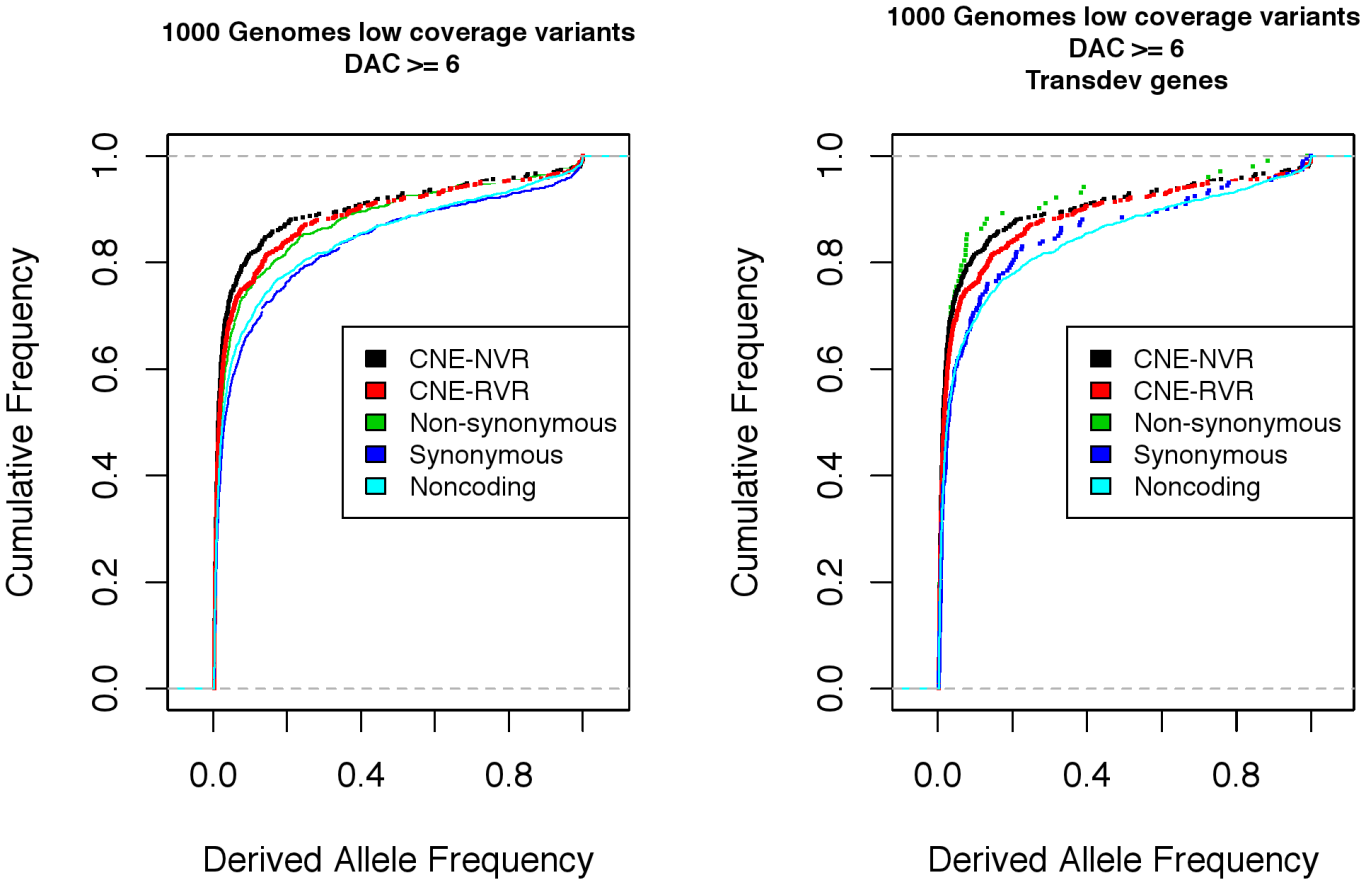
Cumulative derived allele-frequency in CNEs and control regions from 1000 Genomes Project. An excess of rare derived alleles is observed in CNE-NVRs, CNE-RVRs and Non-synonymous sites relative to Synonymous and Non-coding controls (using polymorphic sites with Derived Allele Count (DAC) > = 6). The spectra at CNEs is comparable to the spectra at highly conserved trans-dev genes associated with CNEs.

There are two broad explanations for the excess of substitutions that have occurred in Restricted Variable Regions of CNEs (compared with neighbouring NVRs): they could be neutral (or nearly neutral) changes persisting through genetic drift, or they could be adaptive changes fixed by selection. The first possibility would imply relaxed purifying selection acting on these sites, which should be apparent in greater within-species polymorphism. On the other hand, in the second case, purifying selection could be of comparable strength in the NVRs and RVRs. In order to investigate whether selective constraint in both classes of CNE sites is comparable, we therefore looked at both the proportion of polymorphic sites and spectra of derived allele frequencies. The imputation accuracy for low coverage imputed SNPs in the 1000 Genomes Project was highest for SNPs with an allele count of at least six [31]. Any biases introduced by imputation should affect both classes of CNE sites equally. Therefore, we chose to use this cut-off in our derived allele frequency spectra analyses of CNEs in comparisons with coding sequences. In fact the conclusions of the analysis are unaltered if alleles with lower counts are included: the derived allele frequency spectra obtained taking into account SNPs where the derived allele count is at least one is given in Figure S1. When we consider all observed SNPs in the two CNE categories, there are significantly fewer polymorphisms in NVRs relative to RVRs (Table 2). This observation indicates stronger selective constraint at sites that have been conserved across all lineages suggesting mutations in these regions may have functional consequences. In Figure 1 we observe a significant difference between the derived allele frequency spectra between the two classes of CNE sites, whereby NVRs have an excess of rare derived alleles compared to RVRs. This distinction between the two classes of sites within a CNE indicate that CNEs are composed of sites that are subject to different levels of evolutionary constraint and may have different roles in a regulatory context.

We note that the G+C content varies considerably in the different classes of constrained sites (NVR 35.8%, RVR 41.9%; non-synonymous 48.9% and synonymous 50.7%), and that an increase in G+C content is associated with an increase in heterozygosity in the human genome [32]. This increased heterozygosity results, at least in part, from an increase in the mutation rate brought about by the deamination of C to T at CpG sites where the cytosine is methylated [33]. In agreement with this, the proportion of SNPs occurring at the cytosine in ancestral CpG sites also varies across the different classes of site (NVR 6.7%, RVR 14.7%; non-synonymous 12.3% and synonymous 21.1%). Even after normalising for G+C content, purifying selection at CpG sites appears strongest at NVRs and considerably stronger than at RVRs (normalised values assuming 50% G+C are; NVR 9.35%, RVR 17.5%; non-synonymous 12.6%, synonymous 20.8%) in agreement with overall levels of constraint across the different classes of sites. In addition, all else being equal, the frequency of non-synonymous segregating sites would be slightly depressed, since the frequency of mutations appears to be additionally affected by neighbouring bases, in a manner that reduces non-synonymous mutations [34]; a pattern which might have been shaped by selection to reduce mutational load. Not all conserved non-coding sites show exceptionally low heterozygosity: an elevation in heterozygosity in HapMap and Environmental Genome Project data at a different set of conserved non-coding sites (CNSs), has been attributed to weaker selective effects on CNSs [35]. Similarly, we argue that the higher level of heterozygosity in RVRs suggests a relatively smaller proportion of deleterious mutations than at NVRs and non-synonymous sites.

### Purifying selection in CNEs is more consistent than in coding sequences

Previous studies have shown that UltraConserved Elements (UCEs) defined by human-mouse-rat comparisons experience stronger purifying selection than coding regions [36]. UCEs are (by definition) 100% conserved across at least 200 bp in the three mammalian genomes used to detect them. However, they often overlap exons where non-synonymous mutations can contribute to the excess of rare derived alleles. By contrast, CNEs do not overlap any known exons and represent a larger set of sequences conserved across all jawed vertebrates and with a broader range of sequence identity. Nevertheless,

NVRs within CNEs exhibit a similar proportion of sites that are polymorphic relative to non-synonymous sites (Table 2). The low diversity at NVRs in CNEs is accompanied by a derived allele frequency spectrum that shows a significant excess of rare derived alleles relative to non-synonymous sites (Figure1, Table 3), indicating stronger purifying selection at NVRs. A comparable pattern of polymorphism in mouse ultraconserved elements was interpreted in a similar manner [37]. In a separate study involving human-mouse Conserved Non-Coding sequences (CNCs) an excess of rare derived alleles relative to non-synonymous sites was observed in only those CNCs spanning 5′ and 3′ UTRs (UnTranslated Regions), indicating that conserved sequences in UTRs have an excess of weakly deleterious alleles compared to non-synonymous sites [38].

**Table 3:** *P*-values from Chi-square and K-S tests.

Categories of sites (Derived Allele Count (DAC) > = 6)		χ^2^ *P* value	K-S *P* value
CNE - NVR	CNE - RVR	<2.20E-016	0
CNE - NVR	Non-synonymous	0.2364	0
CNE - NVR	Synonymous	<2.20E-016	7.07E-011
CNE - NVR	Non-coding	<2.20E-016	3.57E-008
CNE - RVR	Non-synonymous	<2.20E-016	0.11
CNE - RVR	Synonymous	0.24	1.65E-008
CNE - RVR	Non-coding	1.37E-011	1.55E-005
Non-synonymous	Synonymous	<2.20E-016	2.06E-005
Non-synonymous	Non-coding	<2.20E-016	0
Synonymous	Non-coding	<2.20E-016	0.01

*P*-values from the χ^2^ test (df = 1) to detect differences in proportion of observed polymorphic sites between the different categories and the Kolmogorov-Smirnov test to detect differences in the derived allele-frequency spectra between categories.

In contrast, our dataset comprised sequences conserved over much longer evolutionary periods (fugu:mammals) and contained approximately 5% UTR sequences. Nevertheless we found evidence for strong purifying selection: there was no significant difference between the observed derived allele frequency spectra in CNE-NVRs (KS test, *p*-value  = 0.9045) and the non-synonymous spectrum of derived alleles at the transcriptional and developmental genes (trans-dev genes) the CNEs are associated with. Strong purifying selection is expected at these trans-dev genes, since they are fundamentally important to vertebrate development and amongst the most highly conserved of all vertebrate genes (Figure 1).

**Figure 2:**
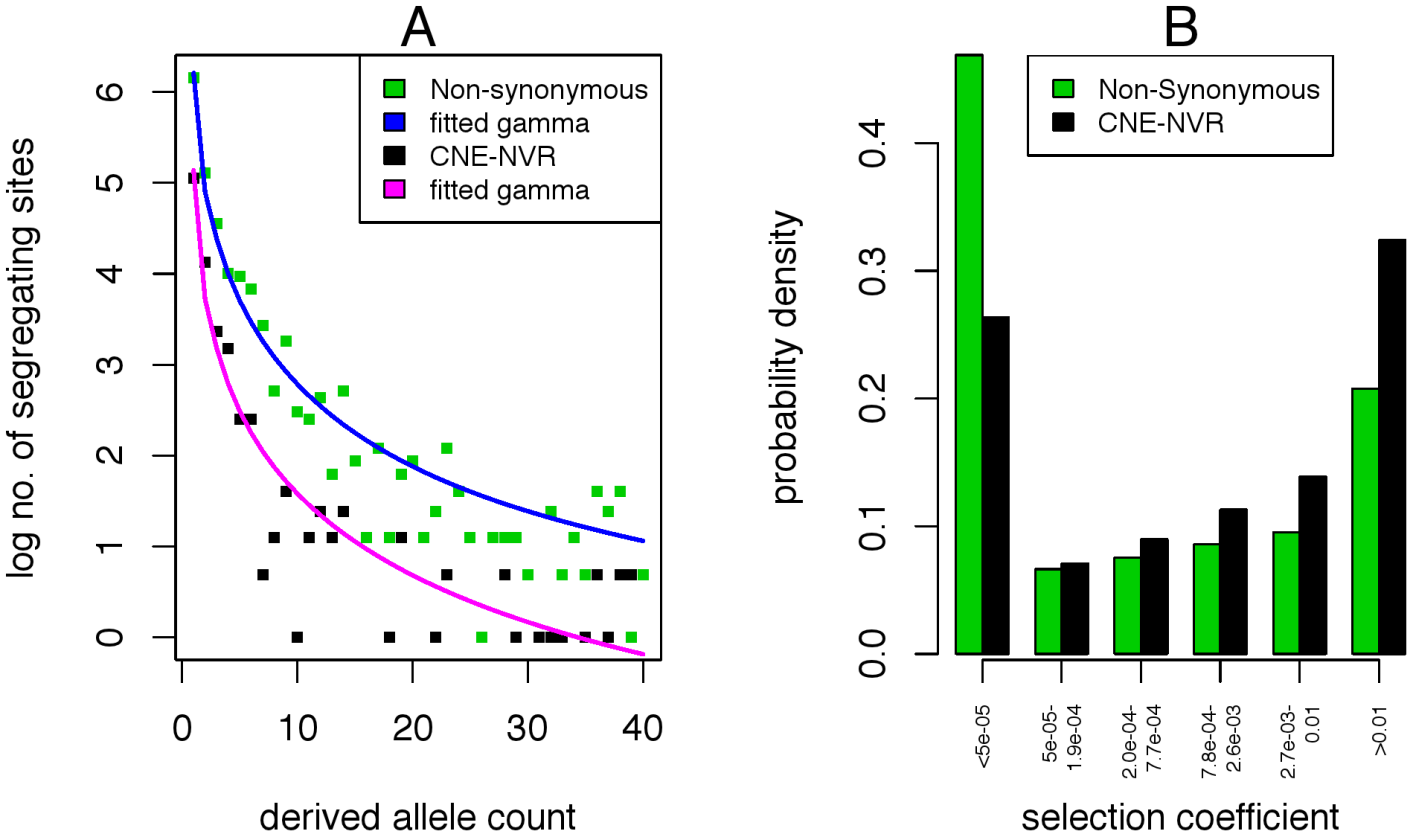
Site-frequency spectra in CNE-NVRs and non-synonymous sites. A) Site-frequency spectra in YRI binned into 3 units. The observed non-synonymous site-frequency spectrum fits a gamma distribution of selective effects (shape  = 0.1, rate  = 6.25). The observed site-frequency spectrum in CNE NVRs fits a gamma distribution of selective effects with a higher mean (shape = 0.18, rate = 7.8). B) The cumulative probability densities of the fitted gamma distributions indicate a larger proportion of lethal sites (>1%) in CNE-NVRs (32%) compared to non-synonymous sites (21%).

We employed the approach of interpreting unfolded site-frequency spectra to infer the distribution of selective effects, as developed for comparisons between synonymous and non-synonymous sites in protein coding regions ([39], [40], [41]). The underlying logic is that mutations under weaker purifying selection have a higher probability of segregating in the population and drifting to higher frequencies than mutations with non-neutral effects. We explored the site-frequency spectra of different classes of sites in the Yoruba population of the 1000 Genomes Project to explain the observed levels of heterozygosity, combined with lower frequency of common alleles found at NVRs, relative to non-synonymous sites. Torgeson et al. [38] report both single estimates and distributions of gamma on the fitness effects of mutations in CNCs. The site-frequency spectrum at non-synonymous sites in our data is best explained by a gamma distribution of selective effects (shape  = 0.1, rate  = 6.25), consistent with previous findings using human polymorphism data [41]. The spectrum for NVRs is best described by a gamma distribution of selective effects with higher mean (shape  = 0.18, rate  = 7.8), with selection sufficient to keep mutations predominantly at lower frequencies (Figure 2A). The probability densities of the fitted gamma distributions (Figure 2B) indicate a larger proportion of lethal sites (selection coefficient |s| >1%) in CNE-NVRs (32%) compared to non-synonymous sites (21%), which suggests that mutations in CNE-NVRs are consistently deleterious.

Since the shape parameter of the gamma distribution of selective effects at non-synonymous sites from the 1000 Genomes data in this study is different from that inferred from previous datasets (e.g. [40]), we used the method of Eyre-Walker et al., 2006 [36] on the allele-frequency spectrum obtained from the 1000 Genomes data for comparison (Table 4). The shape parameter for non-synonymous sites estimated from the method of Boyko et al., 2008 [41] falls within the 95% credibility intervals estimated using the method of Eyre-Walker et al. [40] and is consistent with a larger proportion of effectively neutral mutations in non-synonymous sites relative to CNE-NVRs.

**Table 4:** Distribution of selective effects as inferred by the method of Eyre-Walker et. al, 2006.

	Non-synonymous sites (Eyre-Walker et al., 2006)	Non-synonymous sites	CNE-Nonvariable regions	CNE-Restricted variable regions
Shape	**0.23** (0.19, 0.27)	**0.079** (0.041, 0.17)	**0.19** (0.11, 0.30)	**0.26** (0.13, 0.46)
Mean 4N_e_s	**425** (225, 766)	**65900** (80.8, 475000)	**11840** (387, 92579)	**28.2** (10.7, 71.4)
**N_e_s**	**Proportion of mutations**			
<1 (effectively neutral)	0.19	0.48	0.21	0.42
1–10 (slightly deleterious)	0.14	0.095	0.11	0.31
10–100 (strongly deleterious)	0.23	0.11	0.18	0.25
100–1000 (strongly deleterious)	0.31	0.31	0.5	0.026
1000–10000 (strongly deleterious)	0.13	0.005	0	0

The parameters estimates are accompanied by 95% credibility intervals (in brackets). The shape parameter for non-synonymous sites from the 1000 Genomes data in this study is different from that inferred from previous datasets (e.g. Eyre-Walker et al., 2006). Note that the categories of N_e_s are different to those from the method of Boyko et al., 2009 and the proportion of mutations in the various categories of N_e_s from the two methods are not directly comparable.

The differences in the estimates of the distribution of selective effects could be attributable to differences in ascertainment in the datasets used to estimate its parameters. For example, Eyre-Walker et al. [40] used Environmental Genome Project (EGP) data from 320 genes involved in biologically important pathways (in 90 individuals) while Boyko et al [41] used genome-wide non-synonymous sites (in 19 individuals), both in US populations. We reason that the more varied SNP discovery panel and whole genome sequencing approach used by the 1000 Genomes Project discovered a larger proportion of rare variation, which contributes to greater diversity at non-synonymous sites than observed in previous datasets.

The different patterns of polymorphism observed at CNEs and protein coding loci can be interpreted in terms of their consequences for the molecular biology of the organism carrying the mutations. Non-synonymous changes can have major effects on protein structure and function, particularly through truncation. One might expect that there will be a specific number of non-synonymous changes in any coding sequence that might render the resultant protein completely non-functional, whereas other more conservative changes might have little or no effect on protein function. This would be reflected in a wide spectrum of selective pressures at a limited number of non-synonymous sites, with some being essentially immutable (thus no variant alleles) and others having relatively high derived-allele frequencies. CNEs represent an entirely different form of functional unit, possibly mediating their action through the binding of large numbers of transcription factors. In general, transcription factor binding sites are highly redundant with a rapid turnover rate (reviewed in [42]) but it has been proposed that large *cis*-regulatory modules such as CNEs might be composed of overlapping sets of binding sites thereby imposing a greater evolutionary constraint at each nucleotide position (reviewed in [24]). Mutations in regulatory motifs can cause tangible changes without being lethal. For example, polymorphisms in binding regions have been associated with allele-specific differential binding of RNA polymerase II and nuclear factor κB [43], and functional differences in transcriptional activity [44]. In *Drosophila*, reduced levels of polymorphism have been observed at functional transcription factor binding sites (i.e. transcription factor bound motifs) relative to instances of the same motif outside of the bound region that are not deemed functional [23]. Bound transcription factor-motifs have also been shown to be under stronger purifying selection than unbound motifs [45]. Consequently, some stretches of CNEs are likely to be under strong purifying selection than others.

The site frequency spectrum in CNE RVRs is consistent with a selective effect that is weaker than at CNE NVRs, reflected by the higher level of heterozygosity in RVRs and the higher proportion of derived alleles that drift to high frequencies. The relative relaxation in the selective effect at RVRs implies a difference in functionality. There is evidence that the length of sequence separating functional binding sites is more important than the composition of sequences in some DNA-protein interactions. For example, transcription factor p73 interacts with its half-sites differently in the presence of spacers of various lengths [46]. Therefore, it is possible that a proportion of some RVRs function as spacers that maintain the functional binding sites in NVRs. Alternatively, the more degenerate positions within a binding site could be concentrated in RVRs resulting in a higher tolerance to mutations.

### Discrepancies between the HapMap and 1000 Genomes datasets

**Figure 3:**
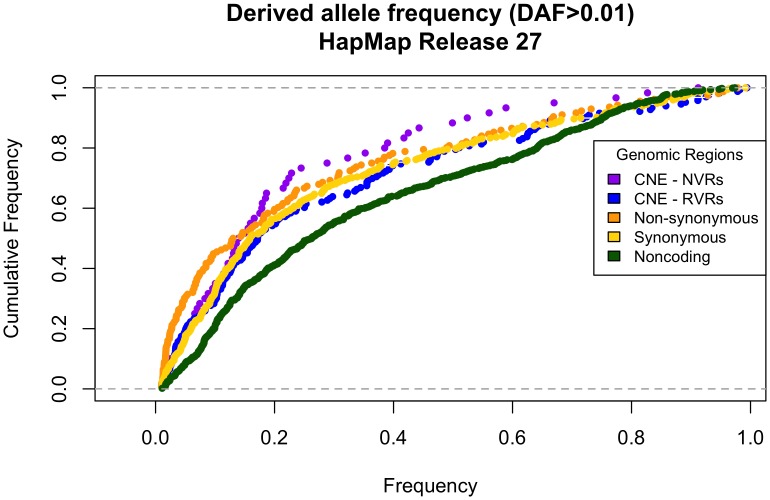
Cumulative derived allele-frequency in CNEs and control regions from the HapMap Project. An excess of rare derived alleles is observed only in Non-synonymous sites relative to Synonymous and Non-coding controls. CNE-NVRs have an excess of rare derived alleles compared to CNE-RVRs however, the derived allele-frequency spectra in CNEs resemble that at synonymous sites as a result of ascertainment bias in the HapMap dataset.

Derived allele frequency spectra from HapMap [47] genotype data have previously been used to compare selective constraint between different types of sequences. For example, Conserved Non-Coding sequences defined by human-mouse and human-dog comparisons, which reflect much smaller divergence times than across the CNEs in this study, have been shown to be under stronger selective constraint than non-conserved regions and under similar constraint to non-synonymous mutations [25]. Before the 1000 Genomes data was publicly available we also looked at the derived allele-frequency spectra from HapMap Release #27. The derived allele frequency spectra of both categories of CNE SNPs obtained from the HapMap Project (Figure 3) are not significantly different to the spectrum at synonymous sites.

These discrepancies reflect a bias in the data toward coding SNPs due to the ascertainment procedure in the HapMap Project, given that it was designed to capture common variants, particularly in or near coding regions. In the HapMap Project, SNPs with a minor allele frequency of >0.05 in a panel of individuals with African, European and Asian ancestry were given preference. Much rare variation that is private to a specific population is lost in this way resulting in a large number of rare variants in non-exonic regions being excluded (reviewed in [48]). Furthermore, selected ENCODE regions were re-sequenced, hence rare variation in these regions are more likely to be captured. Notably, CNEs do not overlap any of the 15 ENCODE regions resequenced in Phases I and III of the HapMap Project. Preference was also given to validated SNPs in the HapMap Pilot Project [47]. Most non-coding variants are unlikely to be validated by other studies given the focus on mutation screening in exomes and there may still be a great number of singletons - i.e. present only in a single copy in the sampled population - being discarded as false positives by rigorous filtering.

With the HapMap dataset it is still possible to observe that non-synonymous sites have an excess of rare derived alleles relative to synonymous sites. Similarly we observe that NVRs in CNEs have an excess of rare derived alleles relative to RVRs. However it is impossible to determine that NVRs in CNEs are under stronger purifying selection than non-synonymous sites because the derived allele frequency spectra at both types of non-coding sequence (conserved and non-conserved) is biased downward relative to the spectra at both types of coding sequence. The results are clearer with the 1000 Genomes dataset because, albeit at low coverage, the whole genome sequencing approach captures a greater number of rare variants outside of the exome.

## Conclusions

Unlike earlier studies, we have focused specifically on deeply conserved, pan-vertebrate, non-coding elements that have high regulatory potential during early development. Given their likely functional roles, we wanted to examine the strength of selective constraint at base pair resolution within these elements. We find different levels of purifying selection, as measured by both evolutionary conservation and human variation, across these large elements and show that some NVR sites are under consistently stronger purifying selection than non-synonymous sites in coding sequences, critically suggesting that mutations in NVRs within CNEs are likely to be deleterious.

The focus on exome-wide sequencing may benefit identifying causal variants in diseases/disorders that follow Mendelian patterns of inheritance [49] [50] [51]. However, the study of complex genetic diseases/disorders, for example developmental disorders determined by perturbation of regulatory networks, warrants either whole genome sequencing or targeted re-sequencing of putative regulatory regions to identify alleles that contribute to an increased risk of occurrence. Variant discovery pipelines in many genome-wide re-sequencing projects discard non-coding variants altogether resulting in potentially important data being lost. Because evolutionarily conserved non-coding DNA represents a small fraction of the vast non-coding landscape, the addition of loci spanning such regions to existing exome selection strategies may be particularly valuable.

We have combined deep, historical phylogenetic footprinting with the occurrence of SNPs and their derived allele frequencies in human populations to identify two classes of sites (Non Variable Regions and Restricted Variable Regions) in CNEs that experience different effects of selective pressure. Increasingly, approaches are combining phylogenetic footprinting with population genomics to effectively identify evolutionarily conserved non-coding sites that are likely to be functional and thus contribute to phenotypic variability and genetic disease [31] [52] [53]. We also found in our analyses that estimates for the distribution of selective effects at non-synonymous sites is different to those inferred from previous datasets, which is possibly attributable to the ascertainment strategy of variants where a whole genome approach across a geographically diverse variant discovery panel results in an unprecedented number of variants being captured.Future work will focus on mapping transcription factor binding sites from ChIP-Seq and other data to CNEs to further explore the relationship between deep evolutionary conservation and binding site degeneracy, paving the way for a better understanding of the role of mutations in regulatory regions in genetic disease. This type of approach has recently proven successful in an analysis of type 2 diabetes risk loci [54]. We might also get a clearer picture of whether overlapping known transcription factor binding sites might explain the signature of unexpectedly strong purifying selection acting on invariant sites in CNEs.

## Methods

### Generating multiple sequence alignments

CNE sequences in fasta format were downloaded from the CONDOR database [14] for the following species: *Homo sapiens, Macaca mulatta*, *Mus musculus*, *Gallus gallus*, *Xenopus tropicalis, Danio rerio and Takifugu rubripes*. Out of ∼7000 human-fugu CNEs spanning a combined length of ∼ 800,000 bp, we identified a subset of 1809 CNEs spanning 318286 bp that could be aligned across all seven species. Clustalw with default parameters was used to align the fasta sequences. Fasta sequences for the seven vertebrate species can be downloaded from the CONDOR website using the list of CNE IDs provided in Text S1.

### Classifying NVRs and RVRs in CNEs

We defined two classes of sites within CNEs based on their conservation across the six vertebrate species by masking the human sequence. Custom Perl scripts were used to distinguish between sites that are identical in all six species (NVRs) and those that vary in at least one species (RVRs). In instances where the presence of the alternate allele in the human reference sequence made a non-variable region a variable region, the site was reclassified as a Non-Variable Region that is polymorphic. There were 27 polymorphic sites reclassified in this way. 161361 bp across 1809 CNEs were non-variable in all seven species while 156925 bp were variable in at least one species.

### Control regions

#### a) Coding

The Biomart [55] tool on Ensembl 71 [56] was used to retrieve the exon coordinates of all transcripts on forward strand genes within 500 kbp of the CNEs in the analysis and the trans-dev genes associated with CNEs according to the CONDOR database. The transcript with the longest coding sequence was retained. The number of 0-fold, 2-fold and 4-fold degenerate sites in the full transcript with the longest coding sequence was obtained by using the software MEGA 5.1 [57]. The relative proportion of non-synonymous sites (0-fold degenerate) in the genic sequences was thus determined to be 64% whereas the proportion of synonymous sites (2 and 4-fold degenerate) was determined to be 36%. The genomic coordinates of the coding sequence for each transcript was used to extract variants. The consequence of the SNP to the transcript was determined from Ensembl VEP (Variant Effect Predictor) web tool [58].

#### b) Non-coding

The non-coding regions for the HapMap analysis only were randomly chosen from five different chromosomes. In the analysis of the 1000 Genomes Project data, the main dataset in this study, genomic regions within 5–6 kb away from the CNEs in the analysis that do not overlap known exons and CNEs constitute the non-coding control. The distance of 5–6 kb was small (corresponding to 0.006 cM in the human genome), so the sites would be exposed to the local reduction in effective population size due to any background selection. Previous analyses (e.g. Hernandez et al., 2011, [59] have shown that these effects extend over ten times that range (>0.08 cM) with a twofold reduction in the levels of diversity being observed beyond genetic distances of 0.043 cM. Any bases in the non-coding controls that overlapped annotated GERP elements were excluded from the analysis.

### Extracting allele frequencies from the HapMap Project

We used the marker IDs of SNPs reported from Biomart to extract the allele frequencies of SNPs from the HapMap Release #27 dataset with a customised XML query. 721 SNPs in ∼800,000 bp of CNE regions were reported from HapMap Release #27. 182 non-synonymous, 400 synonymous and 982 non-conserved non-coding SNPs were obtained from length-matched control regions. The number of SNPs that mapped to 318286 bp is 70 SNPs in CNE NVRs and 176 SNPs in CNE RVRs. The allele frequencies were averaged across all the populations to obtain a global allele frequency for a given variant.

### Extracting variants from 1000 Genomes Project

Variants were extracted from the file; “ftp://ftp.1000genomes.ebi.ac.uk/vol1/ftp/release/20110521/supporting/ALL.wgs.project_consensus_vqsr2b.20101123.snps.low_coverage.sites.vcf.gz” using tabix [60]. The resulting vcf file was parsed using custom Perl scripts to obtain derived allele frequencies of variants. Variants with an alternate allele count of zero in the vcf file were excluded.

The ancestral state of all variants in the analysis was obtained from the vcf files located in; “ftp://ftp.1000genomes.ebi.ac.uk/vol1/ftp/technical/working/20120213_phase1_integrated_release_version1/”. All genomic coordinates were based on Hg19 Feb 2009 assembly of the human genome, Genome Reference Consortium GRCh37.

### Significance testing

Kolmogorov-Smirnov and Chi-squared significance tests were carried out in R [61].

### Base composition

The number of each type of nucleotide and the number of ‘CG’ occurrences were calculated from the fasta sequences of CNEs and coding controls using a combination of Perl and Biopython scripts.

### Site-frequency spectra analyses

We used the full synonymous site-frequency spectra from the YRI population in the 1000 Genomes project as a neutral standard to fit a gamma distribution of selective effects using the population expansion model in the prfreq software [41]. The demographic parameters of tau (scaled time since non-stationary population dynamics) and omega (ratio of ancestral to current population size) derived from African-American data in [41] were not good predictors of the non-synonymous site-frequency spectrum in YRI, reflecting admixture in the African-American data. We found that using a smaller effective population size of Ne  = 12818 predicted the observed site-frequency spectra better, reflecting a relatively milder population expansion in YRI. We then used the estimated demographic parameters under a gamma distribution of selective effects (shape  = 0.1, rate  = 6.25) to fit the observed non-synonymous site-frequency spectrum in YRI. Since there was a significant difference in the observed derived allele-frequency spectra between synonymous sites and non-coding regions in the vicinity of the CNEs, the site frequency spectrum at non-coding sites was used to infer the demographic parameters (tau  = 0.45, omega  = 0.75, Ne  = 16000) for assessing the distribution of selective effects in CNE-NVRs. A gamma distribution of selective effects with a higher mean (shape  = 0.18, rate = 7.8) fitted the observed spectra at CNE-NVRs.

## Supporting Information

Figure S1 **Cumulative derived allele-frequency in CNEs and control regions from 1000 Genomes Project (full spectrum).** An excess of rare derived alleles is observed in CNE-NVRs, CNE-RVRs and Non-synonymous sites relative to Synonymous and Non-coding controls when the full spectrum (Derived Allele Count (DAC) > = 1) of variants is used.
(TIFF)

File S1 **List of CNE accession numbers from the CONDOR database [14].**
(DOC)
