# Supplementary figures and images for: Purifying Selection in Deeply Conserved Human Enhancers Is More Consistent than in Coding Sequences
Source: PLoS One. 2014 Jul 25;9(7):e103357. doi: 10.1371/journal.pone.0103357 (PMC4111549; doi:10.1371/journal.pone.0103357)

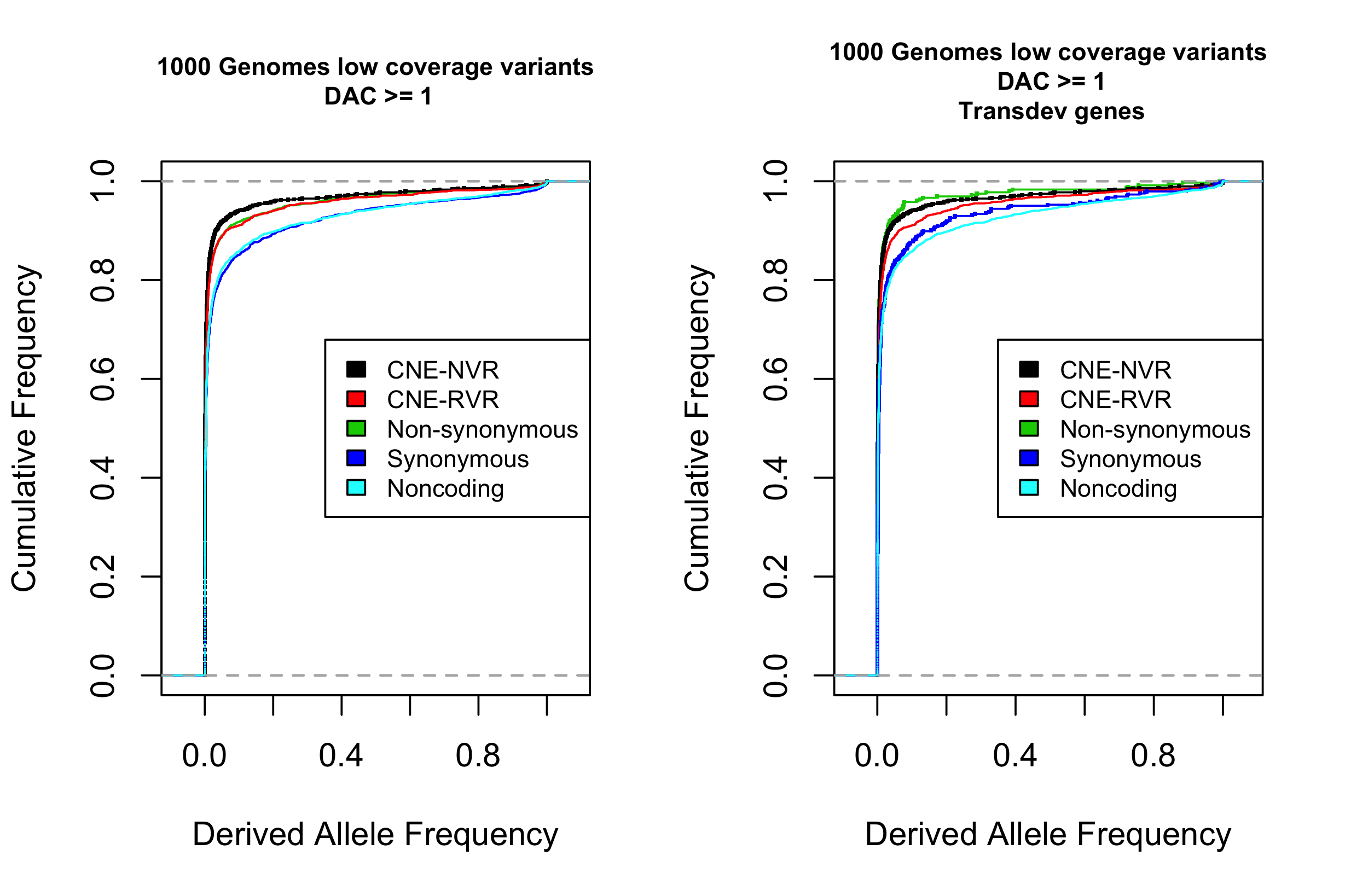

Supplement: Figure S1 — Cumulative derived allele-frequency in CNEs and control regions from 1000 Genomes Project (full spectrum). An excess of rare derived alleles is observed in CNE-NVRs, CNE-RVRs and Non-synonymous sites relative to Synonymous and Non-coding controls when the full spectrum (Derived Allele Count (DAC) > = 1) of variants is used. (TIFF) [file pone.0103357.s001.tiff]
