# Supplementary material for: Purifying Selection in Deeply Conserved Human Enhancers Is More Consistent than in Coding Sequences
Source: PLoS One. 2014 Jul 25;9(7):e103357. doi: 10.1371/journal.pone.0103357 (PMC4111549; doi:10.1371/journal.pone.0103357)
Supplement: File S1 — List of CNE accession numbers from the CONDOR database [14]. (DOC) [file pone.0103357.s002.doc]

CRCNE00004535

CRCNE00002651

CRCNE00001271

CRCNE00008394

CRCNE00010983

CRCNE00005707

CRCNE00003641

CRCNE00008369

CRCNE00011241

CRCNE00005558

CRCNE00000321

CRCNE00003175

CRCNE00006115

CRCNE00011003

CRCNE00000341

CRCNE00005396

CRCNE00010529

CRCNE00011259

CRCNE00005765

CRCNE00008404

CRCNE00000367

CRCNE00009802

CRCNE00008162

CRCNE00001695

CRCNE00004157

CRCNE00003507

CRCNE00006900

CRCNE00001286

CRCNE00010507

CRCNE00010984

CRCNE00003111

CRCNE00009649

CRCNE00008465

CRCNE00004548

CRCNE00009183

CRCNE00007243

CRCNE00008377

CRCNE00007353

CRCNE00003214

CRCNE00004529

CRCNE00009674

CRCNE00005738

CRCNE00009724

CRCNE00008374

CRCNE00001439

CRCNE00008145

CRCNE00000345

CRCNE00003576

CRCNE00004165

CRCNE00001327

CRCNE00008289

CRCNE00003260

CRCNE00007366

CRCNE00010560

CRCNE00001456

CRCNE00008504

CRCNE00008742

CRCNE00003450

CRCNE00009205

CRCNE00002664

CRCNE00008439

CRCNE00005706

CRCNE00001426

CRCNE00003629

CRCNE00009799

CRCNE00005745

CRCNE00003004

CRCNE00004118

CRCNE00001783

CRCNE00001430

CRCNE00011220

CRCNE00008184

CRCNE00005408

CRCNE00001422

CRCNE00003463

CRCNE00006883

CRCNE00001570

CRCNE00001269

CRCNE00005547

CRCNE00001281

CRCNE00002189

CRCNE00007403

CRCNE00009704

CRCNE00006911

CRCNE00004173

CRCNE00001304

CRCNE00001812

CRCNE00009577

CRCNE00005393

CRCNE00004557

CRCNE00003632

CRCNE00009840

CRCNE00003514

CRCNE00009748

CRCNE00001669

CRCNE00000753

CRCNE00003648

CRCNE00006334

CRCNE00000131

CRCNE00003202

CRCNE00008104

CRCNE00001876

CRCNE00010489

CRCNE00003573

CRCNE00008110

CRCNE00009654

CRCNE00008468

CRCNE00007925

CRCNE00008227

CRCNE00003057

CRCNE00003500

CRCNE00011213

CRCNE00003486

CRCNE00003209

CRCNE00001709

CRCNE00000161

CRCNE00010974

CRCNE00001459

CRCNE00005714

CRCNE00007227

CRCNE00008733

CRCNE00002082

CRCNE00000070

CRCNE00002097

CRCNE00001293

CRCNE00003622

CRCNE00003603

CRCNE00008383

CRCNE00009179

CRCNE00004180

CRCNE00008172

CRCNE00003569

CRCNE00006905

CRCNE00002672

CRCNE00006308

CRCNE00006111

CRCNE00007373

CRCNE00009202

CRCNE00011200

CRCNE00003560

CRCNE00005537

CRCNE00008746

CRCNE00003485

CRCNE00001577

CRCNE00000308

CRCNE00003510

CRCNE00008435

CRCNE00007216

CRCNE00010260

CRCNE00003602

CRCNE00008378

CRCNE00003616

CRCNE00001445

CRCNE00006314

CRCNE00008500

CRCNE00008962

CRCNE00003000

CRCNE00001777

CRCNE00004538

CRCNE00007392

CRCNE00008273

CRCNE00000325

CRCNE00010241

CRCNE00008391

CRCNE00010503

CRCNE00008980

CRCNE00001730

CRCNE00008942

CRCNE00006331

CRCNE00007919

CRCNE00003133

CRCNE00002660

CRCNE00010552

CRCNE00002681

CRCNE00005546

CRCNE00002075

CRCNE00008269

CRCNE00003548

CRCNE00004152

CRCNE00004225

CRCNE00007222

CRCNE00007283

CRCNE00001674

CRCNE00007297

CRCNE00008406

CRCNE00009621

CRCNE00001292

CRCNE00006122

CRCNE00006340

CRCNE00010886

CRCNE00008480

CRCNE00006121

CRCNE00001318

CRCNE00011236

CRCNE00003552

CRCNE00008956

CRCNE00009711

CRCNE00004080

CRCNE00009717

CRCNE00000077

CRCNE00008428

CRCNE00003194

CRCNE00008387

CRCNE00000769

CRCNE00009837

CRCNE00008974

CRCNE00003218

CRCNE00000355

CRCNE00005566

CRCNE00000352

CRCNE00003171

CRCNE00008751

CRCNE00003035

CRCNE00003482

CRCNE00003619

CRCNE00001722

CRCNE00006106

CRCNE00003088

CRCNE00011243

CRCNE00005400

CRCNE00009578

CRCNE00010890

CRCNE00003657

CRCNE00001311

CRCNE00007921

CRCNE00001267

CRCNE00004078

CRCNE00007346

CRCNE00001665

CRCNE00008936

CRCNE00005717

CRCNE00003503

CRCNE00003010

CRCNE00003541

CRCNE00011199

CRCNE00009630

CRCNE00002090

CRCNE00009138

CRCNE00003246

CRCNE00008371

CRCNE00008461

CRCNE00009817

CRCNE00001821

CRCNE00002667

CRCNE00000178

CRCNE00001289

CRCNE00003644

CRCNE00010885

CRCNE00000339

CRCNE00008211

CRCNE00004566

CRCNE00003460

CRCNE00007087

CRCNE00006109

CRCNE00004109

CRCNE00006336

CRCNE00006532

CRCNE00001654

CRCNE00008375

CRCNE00004112

CRCNE00004158

CRCNE00009582

CRCNE00008947

CRCNE00008277

CRCNE00004086

CRCNE00010877

CRCNE00004547

CRCNE00007372

CRCNE00003052

CRCNE00007251

CRCNE00008235

CRCNE00008268

CRCNE00006096

CRCNE00000139

CRCNE00004123

CRCNE00003245

CRCNE00008274

CRCNE00003046

CRCNE00003213

CRCNE00006078

CRCNE00003059

CRCNE00008496

CRCNE00009575

CRCNE00003659

CRCNE00007338

CRCNE00003458

CRCNE00007942

CRCNE00002654

CRCNE00009818

CRCNE00009839

CRCNE00006313

CRCNE00007354

CRCNE00006343

CRCNE00009729

CRCNE00005399

CRCNE00003442

CRCNE00003064

CRCNE00003449

CRCNE00008734

CRCNE00008370

CRCNE00005381

CRCNE00000327

CRCNE00001457

CRCNE00000366

CRCNE00006857

CRCNE00010275

CRCNE00011261

CRCNE00003015

CRCNE00007215

CRCNE00009650

CRCNE00003519

CRCNE00001681

CRCNE00011197

CRCNE00010263

CRCNE00007316

CRCNE00003499

CRCNE00011219

CRCNE00005486

CRCNE00006919

CRCNE00010881

CRCNE00008944

CRCNE00008408

CRCNE00008244

CRCNE00001284

CRCNE00002081

CRCNE00000097

CRCNE00007924

CRCNE00008460

CRCNE00001575

CRCNE00002649

CRCNE00003534

CRCNE00003042

CRCNE00003174

CRCNE00002088

CRCNE00004160

CRCNE00006904

CRCNE00003242

CRCNE00001875

CRCNE00009683

CRCNE00003566

CRCNE00008411

CRCNE00008483

CRCNE00001294

CRCNE00003645

CRCNE00003660

CRCNE00004102

CRCNE00001728

CRCNE00003561

CRCNE00008204

CRCNE00003020

CRCNE00003557

CRCNE00000330

CRCNE00003454

CRCNE00008971

CRCNE00005746

CRCNE00003654

CRCNE00009194

CRCNE00008258

CRCNE00009675

CRCNE00008399

CRCNE00003186

CRCNE00010975

CRCNE00008457

CRCNE00003201

CRCNE00000362

CRCNE00001712

CRCNE00004172

CRCNE00008121

CRCNE00006914

CRCNE00001684

CRCNE00003255

CRCNE00006333

CRCNE00008198

CRCNE00005749

CRCNE00001310

CRCNE00007350

CRCNE00008739

CRCNE00001280

CRCNE00001810

CRCNE00006793

CRCNE00007223

CRCNE00008477

CRCNE00009161

CRCNE00008490

CRCNE00006307

CRCNE00011228

CRCNE00006350

CRCNE00001571

CRCNE00002634

CRCNE00011201

CRCNE00005536

CRCNE00009803

CRCNE00007084

CRCNE00007322

CRCNE00003651

CRCNE00007376

CRCNE00007946

CRCNE00010269

CRCNE00005725

CRCNE00009806

CRCNE00009678

CRCNE00001447

CRCNE00001580

CRCNE00004083

CRCNE00008436

CRCNE00000319

CRCNE00003445

CRCNE00006527

CRCNE00008487

CRCNE00001696

CRCNE00006328

CRCNE00003469

CRCNE00003241

CRCNE00008957

CRCNE00003618

CRCNE00009176

CRCNE00000756

CRCNE00010549

CRCNE00000349

CRCNE00000337

CRCNE00003036

CRCNE00003177

CRCNE00005718

CRCNE00003502

CRCNE00002991

CRCNE00001831

CRCNE00008948

CRCNE00001308

CRCNE00004957

CRCNE00003251

CRCNE00003540

CRCNE00008443

CRCNE00005375

CRCNE00008416

CRCNE00000061

CRCNE00008463

CRCNE00001868

CRCNE00003192

CRCNE00008760

CRCNE00001716

CRCNE00000385

CRCNE00009707

CRCNE00010996

CRCNE00001717

CRCNE00005388

CRCNE00004550

CRCNE00004177

CRCNE00007936

CRCNE00001329

CRCNE00000369

CRCNE00000353

CRCNE00005761

CRCNE00007390

CRCNE00001565

CRCNE00007931

CRCNE00008178

CRCNE00003658

CRCNE00003509

CRCNE00002677

CRCNE00001691

CRCNE00008400

CRCNE00008975

CRCNE00008286

CRCNE00003479

CRCNE00005733

CRCNE00008380

CRCNE00008152

CRCNE00002993

CRCNE00011222

CRCNE00004090

CRCNE00008744

CRCNE00003465

CRCNE00007395

CRCNE00011238

CRCNE00003200

CRCNE00007380

CRCNE00009137

CRCNE00000751

CRCNE00002100

CRCNE00008454

CRCNE00003630

CRCNE00003628

CRCNE00001268

CRCNE00003553

CRCNE00008203

CRCNE00008950

CRCNE00009411

CRCNE00003498

CRCNE00005377

CRCNE00000368

CRCNE00004563

CRCNE00008389

CRCNE00001723

CRCNE00009665

CRCNE00004530

CRCNE00006339

CRCNE00002678

CRCNE00003516

CRCNE00001702

CRCNE00007988

CRCNE00006123

CRCNE00001291

CRCNE00008945

CRCNE00005712

CRCNE00010883

CRCNE00008276

CRCNE00008196

CRCNE00005360

CRCNE00005556

CRCNE00004058

CRCNE00003531

CRCNE00004087

CRCNE00009024

CRCNE00005731

CRCNE00000310

CRCNE00003487

CRCNE00003263

CRCNE00004560

CRCNE00001820

CRCNE00003048

CRCNE00004099

CRCNE00003244

CRCNE00009136

CRCNE00003047

CRCNE00006077

CRCNE00010539

CRCNE00005391

CRCNE00008223

CRCNE00003476

CRCNE00000058

CRCNE00010993

CRCNE00003567

CRCNE00005724

CRCNE00001443

CRCNE00008367

CRCNE00008756

CRCNE00000328

CRCNE00008499

CRCNE00003872

CRCNE00009819

CRCNE00005533

CRCNE00001811

CRCNE00005763

CRCNE00007337

CRCNE00001841

CRCNE00003536

CRCNE00003207

CRCNE00000365

CRCNE00006526

CRCNE00005382

CRCNE00000316

CRCNE00005740

CRCNE00008402

CRCNE00000323

CRCNE00003448

CRCNE00006105

CRCNE00010247

CRCNE00005551

CRCNE00010253

CRCNE00002653

CRCNE00009568

CRCNE00008245

CRCNE00004543

CRCNE00010479

CRCNE00011198

CRCNE00010998

CRCNE00007926

CRCNE00009195

CRCNE00010505

CRCNE00001582

CRCNE00004163

CRCNE00010947

CRCNE00004541

CRCNE00008745

CRCNE00003604

CRCNE00005398

CRCNE00008440

CRCNE00006093

CRCNE00008447

CRCNE00009181

CRCNE00009204

CRCNE00006095

CRCNE00003473

CRCNE00009897

CRCNE00010513

CRCNE00002109

CRCNE00004093

CRCNE00003504

CRCNE00001781

CRCNE00003451

CRCNE00007310

CRCNE00002999

CRCNE00008160

CRCNE00008471

CRCNE00005354

CRCNE00007205

CRCNE00008242

CRCNE00004555

CRCNE00007416

CRCNE00001729

CRCNE00009712

CRCNE00009409

CRCNE00009661

CRCNE00006794

CRCNE00007384

CRCNE00000356

CRCNE00003495

CRCNE00003655

CRCNE00010978

CRCNE00003196

CRCNE00004175

CRCNE00004169

CRCNE00001663

CRCNE00009672

CRCNE00008398

CRCNE00008426

CRCNE00008413

CRCNE00010976

CRCNE00008972

CRCNE00001808

CRCNE00008415

CRCNE00009197

CRCNE00005395

CRCNE00002099

CRCNE00003016

CRCNE00003554

CRCNE00001705

CRCNE00008970

CRCNE00005764

CRCNE00002080

CRCNE00000755

CRCNE00010250

CRCNE00001423

CRCNE00010989

CRCNE00006332

CRCNE00010252

CRCNE00003617

CRCNE00009190

CRCNE00005413

CRCNE00004101

CRCNE00001699

CRCNE00003188

CRCNE00003631

CRCNE00001807

CRCNE00003224

CRCNE00007927

CRCNE00001824

CRCNE00007306

CRCNE00006083

CRCNE00006322

CRCNE00007945

CRCNE00005715

CRCNE00008437

CRCNE00000763

CRCNE00006531

CRCNE00009585

CRCNE00010520

CRCNE00009810

CRCNE00008965

CRCNE00007081

CRCNE00003650

CRCNE00007309

CRCNE00002077

CRCNE00009807

CRCNE00011202

CRCNE00004561

CRCNE00008392

CRCNE00008960

CRCNE00001273

CRCNE00003051

CRCNE00009208

CRCNE00007396

CRCNE00004546

CRCNE00001689

CRCNE00006915

CRCNE00008122

CRCNE00002990

CRCNE00004120

CRCNE00001579

CRCNE00008736

CRCNE00003250

CRCNE00009743

CRCNE00008749

CRCNE00008967

CRCNE00003466

CRCNE00005756

CRCNE00011225

CRCNE00003627

CRCNE00004111

CRCNE00006092

CRCNE00010290

CRCNE00010584

CRCNE00005743

CRCNE00001288

CRCNE00008431

CRCNE00008478

CRCNE00003649

CRCNE00008280

CRCNE00011204

CRCNE00009898

CRCNE00003532

CRCNE00003526

CRCNE00005401

CRCNE00009159

CRCNE00004137

CRCNE00003530

CRCNE00002637

CRCNE00001785

CRCNE00008432

CRCNE00007314

CRCNE00009206

CRCNE00005727

CRCNE00003539

CRCNE00005750

CRCNE00004143

CRCNE00003564

CRCNE00005730

CRCNE00001568

CRCNE00008946

CRCNE00008498

CRCNE00003040

CRCNE00008958

CRCNE00007303

CRCNE00000757

CRCNE00008101

CRCNE00002994

CRCNE00008361

CRCNE00007348

CRCNE00009171

CRCNE00003558

CRCNE00005359

CRCNE00006344

CRCNE00009163

CRCNE00008743

CRCNE00003176

CRCNE00004171

CRCNE00010521

CRCNE00008418

CRCNE00002992

CRCNE00009410

CRCNE00000322

CRCNE00004558

CRCNE00002083

CRCNE00006882

CRCNE00009745

CRCNE00003537

CRCNE00008474

CRCNE00010548

CRCNE00001710

CRCNE00009797

CRCNE00011230

CRCNE00004540

CRCNE00010876

CRCNE00001724

CRCNE00007247

CRCNE00003182

CRCNE00005553

CRCNE00006112

CRCNE00004542

CRCNE00004224

CRCNE00008466

CRCNE00004088

CRCNE00008373

CRCNE00008782

CRCNE00000344

CRCNE00011212

CRCNE00003640

CRCNE00003605

CRCNE00002665

CRCNE00008382

CRCNE00010271

CRCNE00006530

CRCNE00001278

CRCNE00004549

CRCNE00007318

CRCNE00008448

CRCNE00004114

CRCNE00002671

CRCNE00008484

CRCNE00007253

CRCNE00000309

CRCNE00005723

CRCNE00008000

CRCNE00009845

CRCNE00008192

CRCNE00003066

CRCNE00001647

CRCNE00011247

CRCNE00006910

CRCNE00004539

CRCNE00005741

CRCNE00008252

CRCNE00008201

CRCNE00007336

CRCNE00007374

CRCNE00006076

CRCNE00005552

CRCNE00004156

CRCNE00004122

CRCNE00001733

CRCNE00010265

CRCNE00000761

CRCNE00006321

CRCNE00001444

CRCNE00006529

CRCNE00010985

CRCNE00000384

CRCNE00007938

CRCNE00005379

CRCNE00004536

CRCNE00003620

CRCNE00008405

CRCNE00009173

CRCNE00005768

CRCNE00000132

CRCNE00001451

CRCNE00001706

CRCNE00004128

CRCNE00005538

CRCNE00011244

CRCNE00008725

CRCNE00002086

CRCNE00008462

CRCNE00005414

CRCNE00001703

CRCNE00007345

CRCNE00009716

CRCNE00007207

CRCNE00009662

CRCNE00000363

CRCNE00005713

CRCNE00007343

CRCNE00001420

CRCNE00011221

CRCNE00003549

CRCNE00010525

CRCNE00001657

CRCNE00004100

CRCNE00006352

CRCNE00008470

CRCNE00002675

CRCNE00009713

CRCNE00003452

CRCNE00000754

CRCNE00007825

CRCNE00004094

CRCNE00009820

CRCNE00000354

CRCNE00010274

CRCNE00003054

CRCNE00008385

CRCNE00008189

CRCNE00008158

CRCNE00009168

CRCNE00009899

CRCNE00003471

CRCNE00003608

CRCNE00000265

CRCNE00003221

CRCNE00003453

CRCNE00008469

CRCNE00008973

CRCNE00003470

CRCNE00008979

CRCNE00000306

CRCNE00011262

CRCNE00008279

CRCNE00003626

CRCNE00008266

CRCNE00003033

CRCNE00010875

CRCNE00007391

CRCNE00011252

CRCNE00011217

CRCNE00004125

CRCNE00004545

CRCNE00003211

CRCNE00009139

CRCNE00006888

CRCNE00003467

CRCNE00008495

CRCNE00003228

CRCNE00004148

CRCNE00003247

CRCNE00004119

CRCNE00008488

CRCNE00004268

CRCNE00007211

CRCNE00009573

CRCNE00003456

CRCNE00001287

CRCNE00001731

CRCNE00001871

CRCNE00005722

CRCNE00008966

CRCNE00008115

CRCNE00002091

CRCNE00009686

CRCNE00004081

CRCNE00010485

CRCNE00007320

CRCNE00009207

CRCNE00009584

CRCNE00009191

CRCNE00008501

CRCNE00005757

CRCNE00005373

CRCNE00002661

CRCNE00009583

CRCNE00000764

CRCNE00008955

CRCNE00003494

CRCNE00004559

CRCNE00009673

CRCNE00006345

CRCNE00000340

CRCNE00008427

CRCNE00007368

CRCNE00002683

CRCNE00003565

CRCNE00008167

CRCNE00006341

CRCNE00001683

CRCNE00008256

CRCNE00008941

CRCNE00005890

CRCNE00007210

CRCNE00002800

CRCNE00007667

CRCNE00010695

CRCNE00007745

CRCNE00006239

CRCNE00006674

CRCNE00007517

CRCNE00009431

CRCNE00005160

CRCNE00002442

CRCNE00011260

CRCNE00011218

CRCNE00008616

CRCNE00005613

CRCNE00000247

CRCNE00006585

CRCNE00002869

CRCNE00005205

CRCNE00006635

CRCNE00007782

CRCNE00005666

CRCNE00005885

CRCNE00010764

CRCNE00000910

CRCNE00010687

CRCNE00005023

CRCNE00006188

CRCNE00007631

CRCNE00009114

CRCNE00003974

CRCNE00003857

CRCNE00002154

CRCNE00010111

CRCNE00006574

CRCNE00008595

CRCNE00007699

CRCNE00003831

CRCNE00004011

CRCNE00006162

CRCNE00003958

CRCNE00002423

CRCNE00001089

CRCNE00002260

CRCNE00000895

CRCNE00009376

CRCNE00007726

CRCNE00005622

CRCNE00002249

CRCNE00003297

CRCNE00003926

CRCNE00003287

CRCNE00002432

CRCNE00009936

CRCNE00005872

CRCNE00006734

CRCNE00004289

CRCNE00000571

CRCNE00000876

CRCNE00002725

CRCNE00003289

CRCNE00005223

CRCNE00002547

CRCNE00007673

CRCNE00010677

CRCNE00000233

CRCNE00001956

CRCNE00006503

CRCNE00009107

CRCNE00005963

CRCNE00007645

CRCNE00007722

CRCNE00001540

CRCNE00002578

CRCNE00009985

CRCNE00001531

CRCNE00010135

CRCNE00011207

CRCNE00008618

CRCNE00003880

CRCNE00003753

CRCNE00002809

CRCNE00002739

CRCNE00000261

CRCNE00001001

CRCNE00000735

CRCNE00009384

CRCNE00003994

CRCNE00003301

CRCNE00006615

CRCNE00004266

CRCNE00007716

CRCNE00004500

CRCNE00010007

CRCNE00007513

CRCNE00000891

CRCNE00003834

CRCNE00006642

CRCNE00009383

CRCNE00006455

CRCNE00002867

CRCNE00002312

CRCNE00007024

CRCNE00006189

CRCNE00006471

CRCNE00007662

CRCNE00006800

CRCNE00009932

CRCNE00009108

CRCNE00007776

CRCNE00005889

CRCNE00001057

CRCNE00009416

CRCNE00000243

CRCNE00000223

CRCNE00007704

CRCNE00005452

CRCNE00006590

CRCNE00005632

CRCNE00003991

CRCNE00010394

CRCNE00009402

CRCNE00003845

CRCNE00006696

CRCNE00011250

CRCNE00003970

CRCNE00004494

CRCNE00000254

CRCNE00007066

CRCNE00005226

CRCNE00007707

CRCNE00004565

CRCNE00003933

CRCNE00006570

CRCNE00008672

CRCNE00005900

CRCNE00000919

CRCNE00001363

CRCNE00009430

CRCNE00010816

CRCNE00004000

CRCNE00002455

CRCNE00004479

CRCNE00009392

CRCNE00009372

CRCNE00006679

CRCNE00000264

CRCNE00005864

CRCNE00007756

CRCNE00004247

CRCNE00000842

CRCNE00003998

CRCNE00000903

CRCNE00000913

CRCNE00006196

CRCNE00003944

CRCNE00005230

CRCNE00000578

CRCNE00010093

CRCNE00007674

CRCNE00007749

CRCNE00002596

CRCNE00009966

CRCNE00011231

CRCNE00005246

CRCNE00002264

CRCNE00004498

CRCNE00009422

CRCNE00001016

CRCNE00000898

CRCNE00008568

CRCNE00000872

CRCNE00003918

CRCNE00004276

CRCNE00010691

CRCNE00007761

CRCNE00003879

CRCNE00001998

CRCNE00008561

CRCNE00006649

CRCNE00002258

CRCNE00007077

CRCNE00009949

CRCNE00009394

CRCNE00006806

CRCNE00000922

CRCNE00009974

CRCNE00005960

CRCNE00005911

CRCNE00000866

CRCNE00007773

CRCNE00010113

CRCNE00003742

CRCNE00008558

CRCNE00007753

CRCNE00006667

CRCNE00003767

CRCNE00010668

CRCNE00005462

CRCNE00006240

CRCNE00006452

CRCNE00001960

CRCNE00001535

CRCNE00001523

CRCNE00006257

CRCNE00007770

CRCNE00000257

CRCNE00005665

CRCNE00000226

CRCNE00002304

CRCNE00009127

CRCNE00009334

CRCNE00002149

CRCNE00003635

CRCNE00000224

CRCNE00002585

CRCNE00001042

CRCNE00008571

CRCNE00007502

CRCNE00005877

CRCNE00003902

CRCNE00010145

CRCNE00000883

CRCNE00003825

CRCNE00000844

CRCNE00007717

CRCNE00007023

CRCNE00003741

CRCNE00000894

CRCNE00010041

CRCNE00002443

CRCNE00009375

CRCNE00003978

CRCNE00005456

CRCNE00009111

CRCNE00006384

CRCNE00009343

CRCNE00008596

CRCNE00007754

CRCNE00003760

CRCNE00010682

CRCNE00006175

CRCNE00005208

CRCNE00006084

CRCNE00003871

CRCNE00005675

CRCNE00003860

CRCNE00007668

CRCNE00005463

CRCNE00001985

CRCNE00009109

CRCNE00003791

CRCNE00006242

CRCNE00003937

CRCNE00007791

CRCNE00010686

CRCNE00000869

CRCNE00009330

CRCNE00003785

CRCNE00003809

CRCNE00010721

CRCNE00000206

CRCNE00008617

CRCNE00006647

CRCNE00002451

CRCNE00005636

CRCNE00011251

CRCNE00002384

CRCNE00002364

CRCNE00004490

CRCNE00005958

CRCNE00006190

CRCNE00008659

CRCNE00006822

CRCNE00009042

CRCNE00000900

CRCNE00007652

CRCNE00003881

CRCNE00002447

CRCNE00011208

CRCNE00000861

CRCNE00001539

CRCNE00006652

CRCNE00007703

CRCNE00001538

CRCNE00005216

CRCNE00007725

CRCNE00006666

CRCNE00003814

CRCNE00009427

CRCNE00001024

CRCNE00005221

CRCNE00003296

CRCNE00002159

CRCNE00007521

CRCNE00005875

CRCNE00002851

CRCNE00002242

CRCNE00005884

CRCNE00002248

CRCNE00002726

CRCNE00009432

CRCNE00006675

CRCNE00011249

CRCNE00009937

CRCNE00005891

CRCNE00011235

CRCNE00007073

CRCNE00002276

CRCNE00000357

CRCNE00003952

CRCNE00000244

CRCNE00004281

CRCNE00003832

CRCNE00001957

CRCNE00000907

CRCNE00001955

CRCNE00009369

CRCNE00007746

CRCNE00003288

CRCNE00000217

CRCNE00004284

CRCNE00008585

CRCNE00005866

CRCNE00006700

CRCNE00005159

CRCNE00006181

CRCNE00000899

CRCNE00000853

CRCNE00004493

CRCNE00002296

CRCNE00002279

CRCNE00002564

CRCNE00002256

CRCNE00004264

CRCNE00007065

CRCNE00007794

CRCNE00007690

CRCNE00001004

CRCNE00009951

CRCNE00009983

CRCNE00005198

CRCNE00011254

CRCNE00003761

CRCNE00000911

CRCNE00002137

CRCNE00003799

CRCNE00003959

CRCNE00003782

CRCNE00003844

CRCNE00009443

CRCNE00001046

CRCNE00009396

CRCNE00000210

CRCNE00003284

CRCNE00001949

CRCNE00000218

CRCNE00008591

CRCNE00002387

CRCNE00000779

CRCNE00007730

CRCNE00000845

CRCNE00006974

CRCNE00009353

CRCNE00001390

CRCNE00002448

CRCNE00006263

CRCNE00003835

CRCNE00010715

CRCNE00009450

CRCNE00006197

CRCNE00009337

CRCNE00000772

CRCNE00007663

CRCNE00006589

CRCNE00005888

CRCNE00009342

CRCNE00000253

CRCNE00004975

CRCNE00003802

CRCNE00008587

CRCNE00003748

CRCNE00009401

CRCNE00001053

CRCNE00000270

CRCNE00006168

CRCNE00006164

CRCNE00005633

CRCNE00000229

CRCNE00010380

CRCNE00005110

CRCNE00005962

CRCNE00010733

CRCNE00000993

CRCNE00004270

CRCNE00006171

CRCNE00010711

CRCNE00001990

CRCNE00007017

CRCNE00003060

CRCNE00006669

CRCNE00003757

CRCNE00006672

CRCNE00002872

CRCNE00006620

CRCNE00004275

CRCNE00007840

CRCNE00005954

CRCNE00006391

CRCNE00005901

CRCNE00000234

CRCNE00007774

CRCNE00000749

CRCNE00006258

CRCNE00009958

CRCNE00007638

CRCNE00003810

CRCNE00004595

CRCNE00000580

CRCNE00008567

CRCNE00003822

CRCNE00011210

CRCNE00000240

CRCNE00007750

CRCNE00006582

CRCNE00008599

CRCNE00006468

CRCNE00003961

CRCNE00001997

CRCNE00003992

CRCNE00005973

CRCNE00006229

CRCNE00006472

CRCNE00003941

CRCNE00000220

CRCNE00000566

CRCNE00010732

CRCNE00001063

CRCNE00004497

CRCNE00010665

CRCNE00004290

CRCNE00011237

CRCNE00007640

CRCNE00000880

CRCNE00011232

CRCNE00001055

CRCNE00003949

CRCNE00010771

CRCNE00002545

CRCNE00002820

CRCNE00004285

CRCNE00004488

CRCNE00004489

CRCNE00006651

CRCNE00009946

CRCNE00007785

CRCNE00001961

CRCNE00003751

CRCNE00007647

CRCNE00002574

CRCNE00009393

CRCNE00000565

CRCNE00004249

CRCNE00005865

CRCNE00010116

CRCNE00000573

CRCNE00005961

CRCNE00002747

CRCNE00005614

CRCNE00007720

CRCNE00005026

CRCNE00010692

CRCNE00007779

CRCNE00010769

CRCNE00009094

CRCNE00003986

CRCNE00006252

CRCNE00009931

CRCNE00009934

CRCNE00000871

CRCNE00003905

CRCNE00000920

CRCNE00006248

CRCNE00006159

CRCNE00003838

CRCNE00001102

CRCNE00007724

CRCNE00002444

CRCNE00009961

CRCNE00011209

CRCNE00005947

CRCNE00007120

CRCNE00002576

CRCNE00005487

CRCNE00010685

CRCNE00006599

CRCNE00007780

CRCNE00010670

CRCNE00000864

CRCNE00002429

CRCNE00005098

CRCNE00002138

CRCNE00000917

CRCNE00007669

CRCNE00006388

CRCNE00006583

CRCNE00005649

CRCNE00001994

CRCNE00009378

CRCNE00000225

CRCNE00006690

CRCNE00000079

CRCNE00009331

CRCNE00003841

CRCNE00000216

CRCNE00000868

CRCNE00003967

CRCNE00006241

CRCNE00005966

CRCNE00007790

CRCNE00000893

CRCNE00010369

CRCNE00005640

CRCNE00005883

CRCNE00003778

CRCNE00001543

CRCNE00004271

CRCNE00005627

CRCNE00001952

CRCNE00003739

CRCNE00004223

CRCNE00007064

CRCNE00004477

CRCNE00004009

CRCNE00010042

CRCNE00003938

CRCNE00009116

CRCNE00003798

CRCNE00003801

CRCNE00000843

CRCNE00005887

CRCNE00006477

CRCNE00007060

CRCNE00006676

CRCNE00007718

CRCNE00000227

CRCNE00005624

CRCNE00003634

CRCNE00010743

CRCNE00002594

CRCNE00002602

CRCNE00002262

CRCNE00001532

CRCNE00007506

CRCNE00000906

CRCNE00009281

CRCNE00007672

CRCNE00002886

CRCNE00009943

CRCNE00009428

CRCNE00003960

CRCNE00007072

CRCNE00002582

CRCNE00007637

CRCNE00009429

CRCNE00009101

CRCNE00010095

CRCNE00001035

CRCNE00007714

CRCNE00005915

CRCNE00003291

CRCNE00009341

CRCNE00007758

CRCNE00001022

CRCNE00004037

CRCNE00006637

CRCNE00003882

CRCNE00011216

CRCNE00005610

CRCNE00009364

CRCNE00003945

CRCNE00009106

CRCNE00003875

CRCNE00000851

CRCNE00000581

CRCNE00003754

CRCNE00003976

CRCNE00005959

CRCNE00005893

CRCNE00006677

CRCNE00009274

CRCNE00010106

CRCNE00010127

CRCNE00002295

CRCNE00001989

CRCNE00009273

CRCNE00009098

CRCNE00001516

CRCNE00003769

CRCNE00006174

CRCNE00011245

CRCNE00005464

CRCNE00000748

CRCNE00004983

CRCNE00003892

CRCNE00005619

CRCNE00007651

CRCNE00006401

CRCNE00002357

CRCNE00003774

CRCNE00005231

CRCNE00009100

CRCNE00003901

CRCNE00008625

CRCNE00003823

CRCNE00006444

CRCNE00009965

CRCNE00001525

CRCNE00007515

CRCNE00001522

CRCNE00003794

CRCNE00009433

CRCNE00003989

CRCNE00001958

CRCNE00001948

CRCNE00009930

CRCNE00000860

CRCNE00003993

CRCNE00010714

CRCNE00007795

CRCNE00009073

CRCNE00000896

CRCNE00010766

CRCNE00010681

CRCNE00002327

CRCNE00000221

CRCNE00011384

CRCNE00004269

CRCNE00003818

CRCNE00003316

CRCNE00000248

CRCNE00007816

CRCNE00011227

CRCNE00007728

CRCNE00002815

CRCNE00004291

CRCNE00004263

CRCNE00006457

CRCNE00000241

CRCNE00002803

CRCNE00005213

CRCNE00000256

CRCNE00003280

CRCNE00004659

CRCNE00003851

CRCNE00000251

CRCNE00002748

CRCNE00005953

CRCNE00007512

CRCNE00010763

CRCNE00009984

CRCNE00006680

CRCNE00010388

CRCNE00002575

CRCNE00006177

CRCNE00008570

CRCNE00007015

CRCNE00001401

CRCNE00009403

CRCNE00001399

CRCNE00000574

CRCNE00001996

CRCNE00000750

CRCNE00008615

CRCNE00003954

CRCNE00007732

CRCNE00004478

CRCNE00006249

CRCNE00001946

CRCNE00005656

CRCNE00004627

CRCNE00008642

CRCNE00007650

CRCNE00003311

CRCNE00005974

CRCNE00004492

CRCNE00002873

CRCNE00000258

CRCNE00000999

CRCNE00003770

CRCNE00009956

CRCNE00006400

CRCNE00002727

CRCNE00009424

CRCNE00000930

CRCNE00009434

CRCNE00004483

CRCNE00008606

CRCNE00011239

CRCNE00000918

CRCNE00007712

CRCNE00000857

CRCNE00000879

CRCNE00001021

CRCNE00003743

CRCNE00002724

CRCNE00003772

CRCNE00004286

CRCNE00010136

CRCNE00003940

CRCNE00005025

CRCNE00009944

CRCNE00001959

CRCNE00009277

CRCNE00000855

CRCNE00000232

CRCNE00003929

CRCNE00003869

CRCNE00005873

CRCNE00002745

CRCNE00010119

CRCNE00001377

CRCNE00005615

CRCNE00001091

CRCNE00001082

CRCNE00005623

CRCNE00010180

CRCNE00010717

CRCNE00005206

CRCNE00001953

CRCNE00010393

CRCNE00000892

CRCNE00009385

CRCNE00002808

CRCNE00001351

CRCNE00003977

CRCNE00009339

CRCNE00006616

CRCNE00005965

CRCNE00009960

CRCNE00003775

CRCNE00006573

CRCNE00002424

CRCNE00003633

CRCNE00009978

CRCNE00003947

CRCNE00004658

CRCNE00007518

CRCNE00003763

CRCNE00007648

CRCNE00007752

CRCNE00007705

CRCNE00002532

CRCNE00010684

CRCNE00003873

CRCNE00007793

CRCNE00006238

CRCNE00010694

CRCNE00003909

CRCNE00009349

CRCNE00004279

CRCNE00003886

CRCNE00004642

CRCNE00006441

CRCNE00009340

CRCNE00005634

CRCNE00005454

CRCNE00000583

CRCNE00003840

CRCNE00006634

CRCNE00001075

CRCNE00007634

CRCNE00005964

CRCNE00003932

CRCNE00005175

CRCNE00003599

CRCNE00002314

CRCNE00001520

CRCNE00001349

CRCNE00009359

CRCNE00009390

CRCNE00006631

CRCNE00009421

CRCNE00004252

CRCNE00000887

CRCNE00006232

CRCNE00007735

CRCNE00005975

CRCNE00011206

CRCNE00009264

CRCNE00000986

CRCNE00009097

CRCNE00007727

CRCNE00007775

CRCNE00001526

CRCNE00008582

CRCNE00005970

CRCNE00007809

CRCNE00006235

CRCNE00001078

CRCNE00004248

CRCNE00008601

CRCNE00009099

CRCNE00006668

CRCNE00010124

CRCNE00003304

CRCNE00004499

CRCNE00007505

CRCNE00010782

CRCNE00002153

CRCNE00003950

CRCNE00007642

CRCNE00009953

CRCNE00002817

CRCNE00003796

CRCNE00007715

CRCNE00007636

CRCNE00005969

CRCNE00002566

CRCNE00002593

CRCNE00003943

CRCNE00000905

CRCNE00002140

CRCNE00005670

CRCNE00003990

CRCNE00007238

CRCNE00000577

CRCNE00009102

CRCNE00000222

CRCNE00006170

CRCNE00005869

CRCNE00001028

CRCNE00011234

CRCNE00007755

CRCNE00006244

CRCNE00008630

CRCNE00010150

CRCNE00006397

CRCNE00000927

CRCNE00002568

CRCNE00000242

CRCNE00007815

CRCNE00002277

CRCNE00007078

CRCNE00004628

CRCNE00009373

CRCNE00002302

CRCNE00009345

CRCNE00008610

CRCNE00006673

CRCNE00001025

CRCNE00004003

CRCNE00003286

CRCNE00006648

CRCNE00011226

CRCNE00003784

CRCNE00009408

CRCNE00009092

CRCNE00002870

CRCNE00007059

CRCNE00002446

CRCNE00005876

CRCNE00010678

CRCNE00006598

CRCNE00009335

CRCNE00009964

CRCNE00005232

CRCNE00006187

CRCNE00010703

CRCNE00006641

CRCNE00002144

CRCNE00000878

CRCNE00005644

CRCNE00007748

CRCNE00010713

CRCNE00001947

CRCNE00002590

CRCNE00003953

CRCNE00003903

CRCNE00002390

CRCNE00000208

CRCNE00000850

CRCNE00006250
